# Groundwater vulnerability to pollution assessment: an application of geospatial techniques and integrated IRN-DEMATEL-ANP decision model

**DOI:** 10.1007/s11356-023-25447-1

**Published:** 2023-02-14

**Authors:** Emmanuel Chibundo Chukwuma, Chris Chukwuma Okonkwo, Oluwasola Olakunle Daniel Afolabi, Quoc Bao Pham, Daniel Chinazom Anizoba, Chikwunonso Divine Okpala

**Affiliations:** 1 Department of Agricultural and Bioresources Engineering, Faculty of Engineering, Nnamdi Azikiwe University, Awka, Nigeria; 2 Research Fellow, Future Africa, University of Pretoria, Pretoria, South Africa; 3 School of Architecture, Building and Civil Engineering, Loughborough University, Loughborough, LE11 3TU UK; 4 Faculty of Natural Sciences, Institute of Earth Sciences, University of Silesia, Katowice, Będzińska Street 60, 41-200, Sosnowiec, Poland

**Keywords:** Groundwater pollution, Decision-making model, Drastic model, GIS, Environmental monitoring

## Abstract

This study evaluated the susceptibility to groundwater pollution using a modified DRASTIC model. A novel hybrid multi-criteria decision-making (MCDM) model integrating Interval Rough Numbers (IRN), Decision Making Trial and Evaluation Laboratory (DEMATEL), and Analytical Network Process (ANP) was used to investigate the interrelationships between critical hydrogeologic factors (and determine their relative weights) via a novel vulnerability index based on the DRASTIC model. The flexibility of GIS in handling spatial data was employed to delineate thematic map layers of the hydrogeologic factors and to improve the DRASTIC model. The hybrid MCDM model results show that net recharge (a key hydrogeologic factor) had the highest priority with a weight of 0.1986. In contrast, the topography factor had the least priority, with a weight of 0.0497. A case study validated the hybrid model using Anambra State, Nigeria. The resultant vulnerability map shows that 12.98% of the study area falls into a very high vulnerability class, 31.90% falls into a high vulnerability, 23.52% falls into the average vulnerability, 21.75% falls into a low vulnerability, and 9.85% falls into very low vulnerability classes, respectively. In addition, nitrate concentration was used to evaluate the degree of groundwater pollution. Based on observed nitrate concentration, the modified DRASTIC model was validated and compared to the traditional DRASTIC model; interestingly, the spatial model of the modified DRASTIC model performed better. This study is thus critical for environmental monitoring and implementing appropriate management interventions to protect groundwater resources against indiscriminate sources of pollution.

**Supplementary Information:**

The online version contains supplementary material available at 10.1007/s11356-023-25447-1.

## Introduction

The importance of groundwater resources to all life forms cannot be over-emphasized, particularly as valuable water resources for irrigation and livestock maintenance (Skevas 2020). Globally, an estimated 1.5 billion people depend on groundwater for various purposes, primarily for drinking (Narsimha and Wu 2019). This dependency on groundwater has led to its overexploitation, which, combined with minimal recharge (due in part to climate change and other factors), has dramatically degraded and depleted groundwater resources (El Alfy et al. 2017). Comparatively, groundwater quality is preferred to surface water due to its natural purification process during water recharge. The depletion and degradation of groundwater resources resulting in mild-to-severe pollution incidents have gained the attention of several researchers (Arya et al. 2020; Aydi 2018). The pollution of groundwater resources has been attributed mainly to anthropogenic activities such as discharging of untreated effluents and chemicals from industries; over-utilization and contamination of fertilizers and pesticides through agricultural activities (Akhtar et al. 2020); and leakages from underground storage tanks, landfills, and septic systems (Ayvaz 2016).

Several studies have shown that groundwater pollution is a significant cause of environmental and health concerns in most developing countries due to poor regulation of anthropogenic activities from industries, agriculture, mining, and waste disposal (Adeyemi and Ojekunle 2021; Ojekunle et al. 2020). Groundwater pollution has dire consequences as it can negatively impact human health, the quality of the environment, and socio-economic development (Li et al. 2021). The prevention and control of groundwater degradation are crucial for effective management due to the complicated nature of the degradation process and the expensive nature of its treatment (Salahu Mohammed Hamza et al. 2014; Sudharshan et al. 2022). Also, assessing groundwater quality and potential is critical for sustainable development (Mahmud et al. 2022; Das and Saha 2022; Azizpour et al. 2021; Sudharshan et al. 2022). Hence, assessing areas vulnerable to groundwater pollution has become an indispensable and effective management tool for groundwater preservation. Geospatial groundwater vulnerability assessment presents a less expensive alternative to conventional groundwater monitoring and management plans. It can also determine risk changes over time due to land-use changes or contaminants migration. This assessment can be easily visualized through maps, utilizing the capability of GIS support. It is, therefore, essential that geospatial map(s) that provides adequate information on the vulnerability of groundwater resource at the national, regional, or local levels be provided periodically to ascertain the state of susceptibility of a given area to pollution. This is indispensable for a region that lacks previous or has scarce records on the vulnerability of groundwater resources, hence the essence of this study.

Vulnerability maps can be used to ensure the implementation of efficient preventive measures to mitigate the pollution of groundwater resources. Vulnerable areas can be mapped using different approaches such as process-based, statistical, overlay, and index methods (Brindha and Elango 2015). Each approach has its advantages and disadvantages. As such, their modification in the form of a hybrid integrated approach, addressing the shortcomings of individual approaches, is inevitable to produce better results. Among the various overlay and index procedures recommended for assessing groundwater vulnerability to pollution, the DRASTIC model has emerged globally as one of the most recognized methods despite some drawbacks to its application (Liang et al. 2016). To provide a reliable assessment of the groundwater vulnerability index or map, the DRASTIC model considers the seven factors of vadose zone impact, topography, soil media, groundwater depth, hydraulic conductivity, aquifer media, and net recharge (Abunada et al. 2020). The model’s simplicity in formulation, flexibility with other factors, and ease of integration with GIS have favored its application over several other models (Abunada et al. 2020). The model also requires less data than other models to describe groundwater vulnerability (Neshat and Pradhan 2017). The DRASTIC model has been extensively applied for the assessment of groundwater vulnerability in various parts of the world, including the USA (Jang et al. 2017), India (Bera et al. 2021; Lathamani et al. 2015), Iran (Moghaddam et al. 2018), Jordan (Khrisat & Al-Bakri 2019), and Algeria (Saida et al. 2017). In Nigeria, it has been applied in groundwater vulnerability assessment in places such as Lagos State (Oladeji 2020), Ondo State (Adewumi et al. 2018), Imo State (Eke et al. 2015; Nnadozie et al. 2019), Abia State (Eke et al. 2015), and Kaduna State (Ahmed et al. 2017).

Despite the wide application of the DRASTIC model, the model has its drawbacks. A major one is the sensitivity of the factors considered by the model, which could vary from region to region (Ouedraogo et al. 2016). Furthermore, uncertainties arise in the process of assigning weights and ratings to the factors of the DRASTIC model by decision-makers (Ouedraogo et al. 2020). To overcome this limitation, multi-criteria decision-making (MCDM) models can be used to obtain weights and ratings of the factors of the DRASTIC model. The MCDM models present a methodological approach to solving a highly complicated decision-making problem (Aydi 2018). These models include methods such as the analytic hierarchy process (AHP) (Costache et al. 2020; Gudiyangada et al. 2020), the analytic network process (ANP) (Gudiyangada et al. 2020), and the Decision-Making Trial and Evaluation and Laboratory (DEMATEL) (Kadoic et al. 2019). To improve the accuracy in the assessment of vulnerability indices, modified MCDM models have been used over the years by combining two models and integrating them with algorithms like interval rough numbers (Wang et al. 2019; Pamucar et al. 2017; Hatefi and Tamošaitienė, 2019), fuzzy sets (Kanani-Sadat et al. 2019), interval-valued fuzzy rough numbers (IVFRN) (Roy et al. 2019), and neutrosophic sets (Nabeeh 2020). Several studies have integrated the MCDM models and the DRASTIC model to enable a more reliable assessment of groundwater vulnerability (Paul and Das 2021; Saida et al. 2017; Torkashvand et al. 2021).

Over the years, researchers have hybridized the MCDM models with other methods to improve the decision-making process’s reliability. To the best of the authors’ knowledge, despite recent applications of hybrid models, the IRN-DEMATEL-ANP model has not been utilized to assess groundwater vulnerability to pollution. Furthermore, there are limited studies on groundwater vulnerability assessment in developing countries, including Nigeria (Omotola et al. 2019; Eugene-Okorie et al. 2020). This study is further critical considering weak water resource management in most developing countries and the necessity for constant environmental monitoring for efficient groundwater management and sustainability. While the MCDM models, such as the analytic hierarchy process (AHP), have been employed in groundwater vulnerability assessment (Goswami and Ghosal 2022; Sahu et al. 2022), a significant limitation relates to inadequacies in capturing uncertainties that might occur in the decision-making process (Hatefi and Tamošaitienė 2019). This limitation constitutes a critical knowledge gap addressed by the novel integration of hybrid models utilized in this study.

Furthermore, the limitation of vulnerability mapping using standard tools such as DRASTIC and EPIK as standalone models has been established. Though these methods account for the spatial variability of groundwater vulnerability, evaluating the essential parameters in their vulnerability assessment (considering their weighting factors) is subjective, requiring expert opinion or engagement using MCDM models (Kassem et al. 2022). This study addresses this shortcoming by integrating expert opinions in the hybridized model. In addition to the contribution of this work, regional-specific criteria and peculiarities were integrated through consultations with local experts while formulating the model. This study aims to provide an improved geospatial model of current knowledge on groundwater vulnerability to environmental pollution by integrating a novel decision model (IRN-DEMATEL-ANP model). Anambra state in Nigeria was used as a case study to validate the model. While studies have been carried out in the state to assess groundwater vulnerability (Emmanuel et al. 2015; Eugene-Okorie et al. 2020), however, to the best of our knowledge, no study in the state has considered the integration of the proposed hybrid MCDM model and the DRASTIC model for a more efficient groundwater vulnerability assessment. The proposed hybrid MCDM model involves the ensemble of the three methods of IRN, DEMATEL, and ANP to provide an accurate and efficient tool for assessing the interactions between several variables and their associated uncertainties. The DEMATEL-ANP ensemble provides an efficient approach for determining the relative importance of variables from a complex multi-criteria decision structure by using matrixes to transform the interdependency of the variables into a causal relationship (Ali et al. 2020). The IRN method further improves the ensemble as the method deals with uncertainty and imprecisions encountered during collective decision-making (Wang et al. 2019). This study seeks to complement and address the limitations of the DRASTIC model as a standalone tool through the integration and hybridization of the MCDM models. This study, therefore, is critical for groundwater management and sustainability. This study provides the scientific basis for water resource management and protection.

## Methodology

This study employed a modified DRASTIC model to assess groundwater pollution using Anambra State as a case study. Seven thematic maps of the DRASTIC model of hydrogeological factors of groundwater depth (D), net recharge (R), aquifer media (A), soil media (S), topography (T), vadose zone impact (I), and hydraulic conductivity (C) were developed. Primary and secondary data were obtained for this study. A geophysical survey using a VES system was employed for primary data collection to determine the resistivity of various soil layers. The data from the geo-electric survey was used to delineate the impact of the vadose zone, the depth-to-water table, hydraulic conductivity, and aquifer media thematic map layers in ArcGIS. Secondary data obtained for this study include rainfall data from the Worldbank’s Climate Database used to delineate net recharge factor, soil data from the Harmonized World Soil Database used to delineate soil media factor, and the Digital Elevation Model (DEM) from the United States Geological Survey used to delineate the topography factor. The weights and ratings of the factors of the DRASTIC model were obtained using a hybrid MCDM model (IRN-DEMATEL-ANP). The model was also used to assess the degree of impact of the factors. Expert opinion obtained in the form of a matrix was used as data input for the model. The IRN was first employed to treat uncertainty and imprecision contained in the expert opinion. The DEMATEL method was then employed to evaluate the significance of the factors and create a relationship network between them. Finally, the ANP method was employed to determine the relative weights of the factors. Based on the determined weights and thematic maps of the factors, the DRASTIC model was employed to present a spatial distribution of the susceptibility to groundwater pollution in the area.

### Study area

**Fig. 1:**
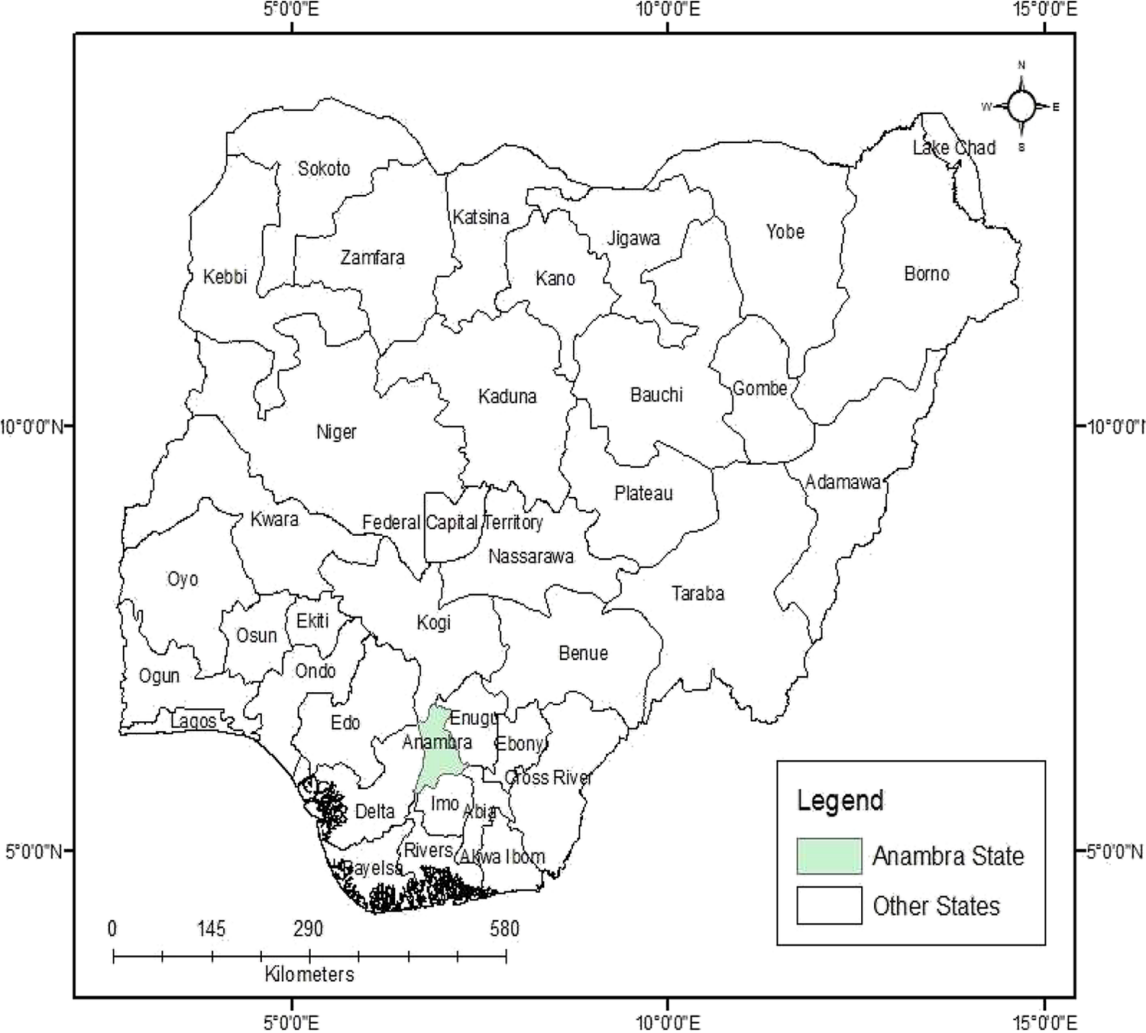
Map of the study area

The study area (Anambra State) is located in the eastern part of Nigeria and lies between latitudes 5° 40ʹ and 6° 48ʹ north and longitudes 6° 35ʹ and 7° 50ʹ east. The state is located in the tropical rain-forest zone of West Africa (Fig. 1) with an average humidity of 80%, a mean daily temperature of 20 °C (Umeh et al. 2020), and a mean annual precipitation of about 2000 mm (Ekenta et al. 2015; Umeh et al. 2020). The state has two main climatic variations: the rainy season, which usually starts in April and ends in September, and the dry season, which begins in October and ends in March (Enekwechi 2017). During the rainy season, the state experiences violent showers, and due to poor water management, surface runoff leads to environmental hazards such as flooding, erosion, and landslides (Enekwechi 2017). Geologically, the state has five predominant formations, including the Imo Formation (Imo shale and Ebenebe sandstone) (Paleocene), the Nsukka Formation (Maastrichtian–Danian), the Ogwashi-Asaba Formation (Oligocene–Miocene), the Ameki Formation (Nanka sandstone and Nsugbe sandstone) (Eocene), and the Benin Formation (Pliocene-Recent) (Anizoba et al. 2020). Hydrologically, the state has surface water sources from springs, streams, lakes, and other major rivers, such as the Anambra River, which serves as a tributary to the Niger River, and the Niger River that connects to the Atlantic Ocean (Egboka and Okoyeh 2019).

### Data collection and analysis

A geophysical survey was conducted to determine the resistivity of various soil layers in the study area. The survey was aimed at determining the variations in resistivity with soil depth. A geo-electrical method that utilizes a vertical electrical sounding (VES) system was employed for the survey. The VES was conducted by deploying an ABEM Terrameter Self-Averaging System (SAS) 1000C device. The instrument can display apparent resistivity values calculated from Ohm’s law digitally. It also automatically records the voltage and current, piles up the results, and determines the resistance in real-time with an instant digital read-out (Chukwuma et al. 2020). A GPS device was used to determine the spatial location and elevation of the various VES points. From the data obtained in the field survey, the apparent resistivity of various soil layers was computed and interpreted with the aid of computer software (one-dimensional Interpex Version 3). The data from the geo-electric survey was used to compute the depth-to-water table. The depth-to-water table and the resistivity of the various soil layers obtained were imputed into an Excel spreadsheet and subsequently exported into the GIS environment. The impact of the vadose zone, a parameter that describes the effect of the unsaturated zone above the groundwater table, was modeled as the apparent resistivity of the last soil layer before the groundwater table. The aquifer media, a parameter that describes the influence of water-bearing rocks or soil, was modeled as the apparent resistivity of the saturated soil layer. Furthermore, the resistivity of the saturated layer was used to calculate the hydraulic conductivity of the study area via Eq. (1):

1 $${K}_{C}={~}^{1}\!\left/ \!{~}_{\rho }\right.$$

where ${K}_{C}$ represents hydraulic conductivity calculated, and $\rho$ represents the resistivity of the saturated soil layer.

Based on the predetermined spatial locations (longitude and latitude), the aforementioned depth-to-water table values, resistivity values, and the calculated hydraulic conductivity values were imported into the GIS software. The IDW was subsequently applied for interpolation to delineate the impact of the vadose zone, the depth-to-water table, hydraulic conductivity, and aquifer media thematic map layers.

The soil data were derived from the Food Agriculture Organization (FAO) database, precisely the Harmonized World Soil Database (HWSD). After geo-referencing and digitization, the soil data was converted to a raster format to delineate the thematic map layer for soil media. The soil media factor describes the influence of the uppermost part of the soil layer, the topsoil. The satellite imagery of the Shuttle Radar Topography Mission (SRTM) was sourced from the United States Geological Survey (USGS) website, and the data was used to define the slope using the surface tool in ArcGIS software. The slope represents the topography as a factor in the DRASTIC model. The mean monthly precipitation data were obtained from World Bank’s climate database for 26 years (1991–2016). The net recharge, a factor that describes the amount of water per unit area that penetrates the ground surface and percolates down to the water table, was calculated as 12% of the average annual precipitation (Eke et al. 2015). The computed net recharge was imported into the GIS environment. IDW tool was used for interpolation to produce net recharge spatial distribution.

### Reclassification and standardization

The map layers of all hydrogeologic factors were standardized using the fuzzy membership system. For conformity among the factors, the standardization of the maps was done to provide a platform for their incorporation. The fuzzy large function was applied when it is considered that a high input value of a given factor is more likely to produce an environmental hazard. For the fuzzy MS small function, this was applied when it is considered that a small input value of a given factor is more likely to produce an environmental hazard (Okonufua et al. 2019).

### Groundwater vulnerability assessment model

The proposed model in this study involves the integration of a hybrid MCDM model and the DRASTIC model in the GIS environment. This study aims to take advantage of the unique capacity of GIS applications in managing geospatial data and the ability of the MCDM model to handle complex decision-making problems (Haroon and Muhammad 2022). A hybrid MCDM model that consists of IRN, DEMATEL, and ANP was used to compute the weight and model the hydrogeologic impact. In the first stage of the proposed assessment model, the IRN-DEMATEL method is used to produce a relationship network between the hydrogeologic factors and determine their influence on groundwater vulnerability. The second step involves using the DEMATEL output to calculate the final weight of the hydrogeologic factors based on the ANP method. In the last step, the DRASTIC model integrates the hydrogeologic factors based on their weights. Further description of the integration of the models, their order of integration, and the DRASTIC model’s output are stated below.

This study’s proposed hybrid MCDM model integrates the IRN, DEMATEL, and ANP methods for decision-making analysis. The IRN method is helpful in the theory of rough sets, handling uncertainty and imprecision in collective decision-making (Wang et al. 2019). It eliminates subjectivity in decision-making problems (Pamucar et al. 2018) by defining uncertain number intervals; its application helps to eliminate the need for additional information in decision-making (Wang et al. 2019). The DEMATEL method is a practical approach for modeling causal dependencies among factors and visualizing their complex cause-and-effect relationships comprehensibly (Pamucar et al. 2017). By using matrices or graphs, the DEMATEL method depicts the relations between various factors of a system in such a way that numbers can be used to describe the intensity of the relationships (Kanani-Sadat et al. 2019). As a result, this method presents a sum of all direct and indirect effects of the various factors transferred to and received by other factors (Wang et al. 2019). The ANP method, proposed by Saaty in 1996 (Mirhosseini et al. 2020), is a generalized form of the AHP method that models decision-making problems as networks instead of hierarchies (Kadoic et al. 2019). It substitutes single-direct relationships with feedback and dependency; the ANP method not only considers linear relationships between factors but also validates their interactions (Matin et al. 2020).

#### Application of the IRN method

The first phase of the hybrid MCDM model is applying the IRN method to treat uncertainty and subjectivity contained in the expert’s decision. The required input data for this phase is the initial pairwise comparison matrix which experts provide. Here, experts provide a bundle of pairwise comparisons between every two distinct factors. A pair of real numbers $\left({l}_{ij}^{k}, {m}_{ij}^{k}\right)$ is used for evaluation, where *k* denotes the number of corresponding experts in which *k* = 1, …, *N*. The judgment of the *k*-th expert about the influence of the *i*-th criterion on the *j*-th one is $\left({l}_{ij}^{k}, {m}_{ij}^{k}\right)$. A predefined scale of real numbers ranging from 0 to 5 was used for evaluation, as shown in Table 1. If a *K* expert is uncertain about the influence between a pair of factors, then ${l}_{ij}^{k}\ne {m}_{ij}^{k}$ is established. On the other hand, if the *K* expert is certain about the decision, then ${l}_{ij}^{k}={m}_{ij}^{k}$ is established. For this study, questionnaires were distributed to four (4) experts with extended years of experience in groundwater study.

**Table 1:** Linguistic values and their corresponding real numbers

Linguistic values	Real numbers
Very high influence (VH)	5
High influence (H)	4
Medium influence (M)	3
Low influence (L)	2
Very low influence (VL)	1
No influence (NO)	0

The obtained pair of real numbers $\left({l}_{ij}^{k}, {m}_{ij}^{k}\right)$ contained in the initial pairwise comparison matrix was then transformed into rough sequences $RN({l}_{ij}^{k}), RN{(m}_{ij}^{k})$, where $RN\left({l}_{ij}^{k}\right)= \left[\underline{Lim} {(l}_{ij}^{k}), \overline{Lim} {(l}_{ij}^{k})\right]$, and $RN{(m}_{ij}^{k})= \left[\underline{Lim} ({m}_{ij}^{k}), \overline{Lim}{ (m}_{ij}^{k})\right]$. $\underline{Lim} {(l}_{ij}^{k})$ and $\underline{Lim} {(m}_{ij}^{k})$ represent the lower limits, and $\overline{Lim} {(l}_{ij}^{k})$ and $\overline{Lim} {(m}_{ij}^{k})$ represent the upper limit of the rough sequences $RN\left({l}_{ij}^{k}\right)$ and $RN{(m}_{ij}^{k})$. For more information about the IRN, see Pamucar et al. (2018), Sepehri et al. (2020), and Stevic et al. (2019).

The next step in the IRN method is aggregating the rough sequence of all the decision-makers, which is done by applying Eqs. (2) and (3).

2 $$RN\left({\overline{l}}_{ij}\right)=RN\left({l}_{ij}^{1}, {l}_{ij}^{2}, \dots , {l}_{ij}^{k}\right) \left\{\begin{array}{c}{\overline{l}}_{ij}^{L}= \frac{1}{M}{\sum }_{k=1}^{M}{l}_{ij}^{kL}\\ {\overline{l}}_{ij}^{U}= \frac{1}{M}{\sum }_{k=1}^{M}{l}_{ij}^{kU}\end{array}\right.$$

3 $$RN\left({\overline{m}}_{ij}\right)=RN\left({m}_{ij}^{1}, {m}_{ij}^{2}, \dots , {m}_{ij}^{k}\right) \left\{\begin{array}{c}{\overline{m}}_{ij}^{L}= \frac{1}{M}{\sum }_{k=1}^{M}{m}_{ij}^{kL}\\ {\overline{m}}_{ij}^{U}= \frac{1}{M}{\sum }_{k=1}^{M}{m}_{ij}^{kU}\end{array}\right.$$

where *k* represents the *k*th expert (*k* = 1*,* 2*,*…*, N*), $RN\left({\overline{l}}_{ij}\right)$ and $RN\left({\overline{m}}_{ij}\right)$ represent the rough sequences that together make up IRN ${\overline{\overline{d}}}_{ij}$ = [(${\overline{l} }_{ij}^{L}$, ${\overline{l} }_{ij}^{U}$), (${\overline{m} }_{ij}^{L}$, ${\overline{m} }_{ij}^{U}$)]. Hence, we obtain the IRN decision matrix as $\overline{\overline{D}}$

4 $$\overline{\overline{D}}={ \left[\begin{array}{cccc}{\overline{\overline{d}}}_{11}& {\overline{\overline{d}}}_{12}& \cdots & {\overline{\overline{d}}}_{1c}\\ {\overline{\overline{d}}}_{21}& {\overline{\overline{d}}}_{22}& \dots & {\overline{\overline{d}}}_{2c}\\ \vdots & \vdots & \ddots & \vdots \\ {\overline{\overline{d}}}_{c1}& {\overline{\overline{d}}}_{c2}& \cdots & {\overline{\overline{d}}}_{cc}\end{array}\right]}_{c\times c}$$

where *c* denotes the number of criteria (hydrogeologic factors).

The last step of this method is the transformation of interval rough numbers into crisp numbers to obtain our initial decision matrix. Hence, the matrix $\overline{\overline{D}}$ is transformed using Eqs. (5) and (6) to obtain the elements of our initial decision matrix $D$ (Eq. (7)).

5 $$\gamma = \left[ \frac{RB\left({\overline{m}}_{ij}\right)}{RB\left({\overline{m}}_{ij}\right)+RB\left({\overline{l}}_{ij}\right)}\right],0\le \gamma \le 1; RB\left({\overline{m}}_{ij}\right)= \left[{{\overline{m} }_{ij}^{U}-\overline{m} }_{ij}^{L}\right]; RB\left({\overline{l}}_{ij}\right)= \left[{{\overline{l} }_{ij}^{U}-\overline{l} }_{ij}^{L}\right]$$

6 $${D}_{ij}= \left[\gamma . {\overline{l} }_{ij}^{L}\right]+ \left[\left(1-\gamma \right).{\overline{m} }_{ij}^{U}\right]$$

7 $$D={ \left[\begin{array}{cccc}{D}_{11}& {D}_{12}& \cdots & {D}_{1c}\\ {D}_{21}& {D}_{22}& \dots & {D}_{2c}\\ \vdots & \vdots & \ddots & \vdots \\ {D}_{c1}& {D}_{c2}& \cdots & {D}_{cc}\end{array}\right]}_{c\times c}$$

#### Application of the DEMATEL method

The second phase of the hybrid MCDM model involves the application of the DEMATEL method to analyze the structure and the strength of the relationships between the factors. The initial decision matrix *D* obtained using the IRN method is used as the input data for the DEMATEL method. The first step of this method involves the normalization of the elements of the initial decision matrix $D$ to obtain the normalized matrix $\overline{\overline{Z}}$, as shown in Eq. (8).

8 $$\overline{\overline{Z}}={ \left[\begin{array}{cccc}{\overline{\overline{z}}}_{11}& {\overline{\overline{z}}}_{12}& \cdots & {\overline{\overline{z}}}_{1c}\\ {\overline{\overline{z}}}_{21}& {\overline{\overline{z}}}_{22}& \dots & {\overline{\overline{z}}}_{2c}\\ \vdots & \vdots & \ddots & \vdots \\ {\overline{\overline{z}}}_{c1}& {\overline{\overline{z}}}_{c2}& \cdots & {\overline{\overline{z}}}_{cc}\end{array}\right]}_{c\times c}$$

The elements ${\overline{\overline{z}}}_{ij}$ of the normalized matrix $\overline{\overline{Z}}$ are obtained using Eqs. (9) and (10).910

The second step in this phase involved evaluating the total relationship between the hydrogeologic factors as illustrated by the matrix $T$. The matrix *T* illustrates the direct/indirect relationships of the hydrogeologic factors and is defined using Eqs. (11) and (12), where *I* is an identity matrix.

11 $$T={ \left[\begin{array}{cccc}{t}_{11}& {t}_{12}& \cdots & {t}_{1c}\\ {t}_{21}& {t}_{22}& \dots & {t}_{2c}\\ \vdots & \vdots & \ddots & \vdots \\ {t}_{c1}& {t}_{c2}& \cdots & {t}_{cc}\end{array}\right]}_{c\times c}$$

12 $${t}_{ij} = {\overline{\overline{z}}}_{ij}\times {\left(I- {\overline{\overline{z}}}_{ij}\right)}^{-1}$$

After determining the total relationship between the conditioning factors, values of $R$ and $S$ were calculated. The value $R$ elucidates the direct/indirect impacts that condition *i* has on other conditions; this is obtained by calculating the sum of the *i*-th row of the matrix $T$ (see Eq. (13)). $S$ demonstrates the general influences of all the criteria on the *j* criterion, and this is obtained by calculating the sum of the *j*-th column of the matrix $T$ (see Eq. (14)).

13 $$R= {\left[{R}_{i}\right]}_{c\times 1}=\sum_{j=1}^{c}{t}_{ij}$$

14 $$S= {\left[{S}_{j}\right]}_{1\times c}=\sum_{i=1}^{c}{t}_{ij}$$

The values $R+ S$ and $R-S$ represent the significance of the criteria and understand the causal relationship between criteria. $R+S$ specifies the measure of influence *i* criterion that has on the remaining criteria and designates its position in the problem. $R-S$ indicates the influence of the criteria in the system, with a positive value demonstrating that the *i*-th criterion is effective and falls into the category of “causes.” Also, a negative value of $R-S$ shows that the *i*-th criterion will be under the influence of others and fall into the category of “effects.” Criteria with a high value of $R-S$ have higher priority, while those with low values have a lower priority.

#### Application of the ANP method

The third and final phase of the hybrid MCDM model involves applying the ANP method to determine the relative weights of the hydrogeologic factors. The total relationship matrix *T* obtained in the model’s previous phase is used as the input data for this phase. The first step in this phase is the creation of an unweighted supermatrix from the total relationship matrix $T$. Furthermore, to obtain the unweighted supermatrix, experts define an α-cut threshold to filter out the minor influences from the matrix $T$. A general schema of α-cut total-relation matrix ${T}_{\propto }$ is shown in Eq. (15). If ${t}_{ij}< \propto$, then ${{t}^{\propto }}_{ij}$ = 0; otherwise, ${{t}^{\propto }}_{ij}= {t}_{ij}$, where ${t}_{ij}$ is the element of the *total*-relation matrix $T$.

15 $${T}^{\propto }= \left[\begin{array}{ccccc}{t}_{11}^{\propto }& \cdots & {t}_{1j}^{\propto }& \cdots & {t}_{1c}^{\propto }\\ \vdots & \vdots & \vdots & & \\ {t}_{i1}^{\propto }& \cdots & {t}_{ij}^{\propto }& \cdots & {t}_{ic}^{\propto }\\ \vdots & \vdots & \vdots & & \\ {t}_{c1}^{\propto }& \cdots & {t}_{cj}^{\propto }& \cdots & {t}_{cc}^{\propto }\end{array}\right]$$

The matrix ${T}^{\propto }$ gives us our unweighted supermatrix. The second step in this method involves the definition of a weighted supermatrix by normalizing the unweighted supermatrix ${T}^{\propto }$. To achieve normalization, the sum of elements of the matrix ${T}^{\propto }$ by columns is determined. The normalization of the matrix ${T}^{\propto }$ yields the elements of the weighted supermatrix $\widetilde{W}$, and the equation is shown as follows:

16 $$\widetilde{W}={ \left[\begin{array}{cccc}{\widetilde{w}}^{11}& {\widetilde{w}}^{12}& \cdots & {\widetilde{w}}^{1c}\\ {\widetilde{w}}^{21}& {\widetilde{w}}^{22}& \dots & {\widetilde{w}}^{2c}\\ \vdots & \vdots & \ddots & \vdots \\ {\widetilde{w}}^{c1}& {\widetilde{w}}^{c2}& \cdots & {\widetilde{w}}^{cc}\end{array}\right]}_{c\times c}$$

where ${\widetilde{W}}^{ij}= {{t}^{\propto }}_{ij}/{\widetilde{d}}_{i}$, and the value of ${\widetilde{d}}_{i}$ is obtained from ${\widetilde{d}}_{i}={\sum }_{i=1}^{c}{{t}^{\propto }}_{ij}$.

The final step of this method involves the determination of the weight of the hydrogeologic factors. Here, a limit supermatrix is obtained by multiplying the weighted supermatrix by itself multiple times. The weighted supermatrix can be raised to the limiting powers until the supermatrix has converged and become a long-term stable supermatrix to obtain global priority vectors, called IRN-DEMATEL-ANP influence weights, such as $\underset{k\to \infty }{\mathrm{lim}}= {\widetilde{W}}^{k}$, where W denotes the limit supermatrix and *k* denotes any number. After determining the individual weights of the hydrogeologic factors, the DRASTIC model was applied to aggregate them and produce a groundwater vulnerability map of the study area.

#### Application of the DRASTIC model

The DRASTIC model is an empirical model which relies on hydrogeologic data to assess the vulnerability of the groundwater systems (Shah et al. 2021). The model is based on four significant assumptions viz:

- introduction of contaminant to the ground *surface* region,
- precipitation is responsible for the transportation of pollutants to groundwater,
- the contaminants have enough water mobility to reach the water table, and
- the area under study is 100 acres or more (Bera et al. 2021; Mondal et al. 2019).

The model can be employed using GIS and hydrogeologic data to delineate thematic map layers representing the DRASTIC model’s factors. The model’s product is an index map of groundwater vulnerability obtained by assigning ratings and weights to the hydrogeologic factors (Jang et al. 2017). A general equation of the DRASTIC model is shown in Eq. (17):

17 $$DI= {D}_{r}{D}_{w}+{R}_{r}{R}_{w}+{A}_{r}{A}_{w}+{S}_{r}{S}_{w}+{T}_{r}{T}_{w}+{I}_{r}{I}_{w}+{C}_{r}{C}_{w}$$

where *DI* represents the DRASTIC index, and *D*, *R*, *A*, *S*, *T*, *I*, and *C* represents the seven hydrogeologic factors of groundwater depth, net recharge, aquifer media, soil media, topography, the impact of the vadose zone, and the hydraulic conductivity respectively. The subscript “*r*” represents the rating of the factors, which was conducted in this study using the fuzzy membership function. The subscript “*w*” represents the relative weight of the factors obtained in this study using the IRN-DEMATEL-ANP model. The raster calculator function was used to run the DRASTIC model and realize the groundwater vulnerability map of the study area.

## Results and discussions

This study modified the DRASTIC model for a more efficient and reliable assessment of groundwater vulnerability. The conventional method of assigning weight and ratings in the DRASTIC model creates subjectivity and uncertainty in the decision-making. Hence, the method of assigning weight employed in this study considers the subjectivity and uncertainty of the decision-making process to produce a more efficient vulnerability map. The seven hydrogeologic factors of the DRASTIC model were delineated with the aid of GIS and standardized using the fuzzy membership system.

### Delineation of hydrogeologic factors

**Fig. 2:**
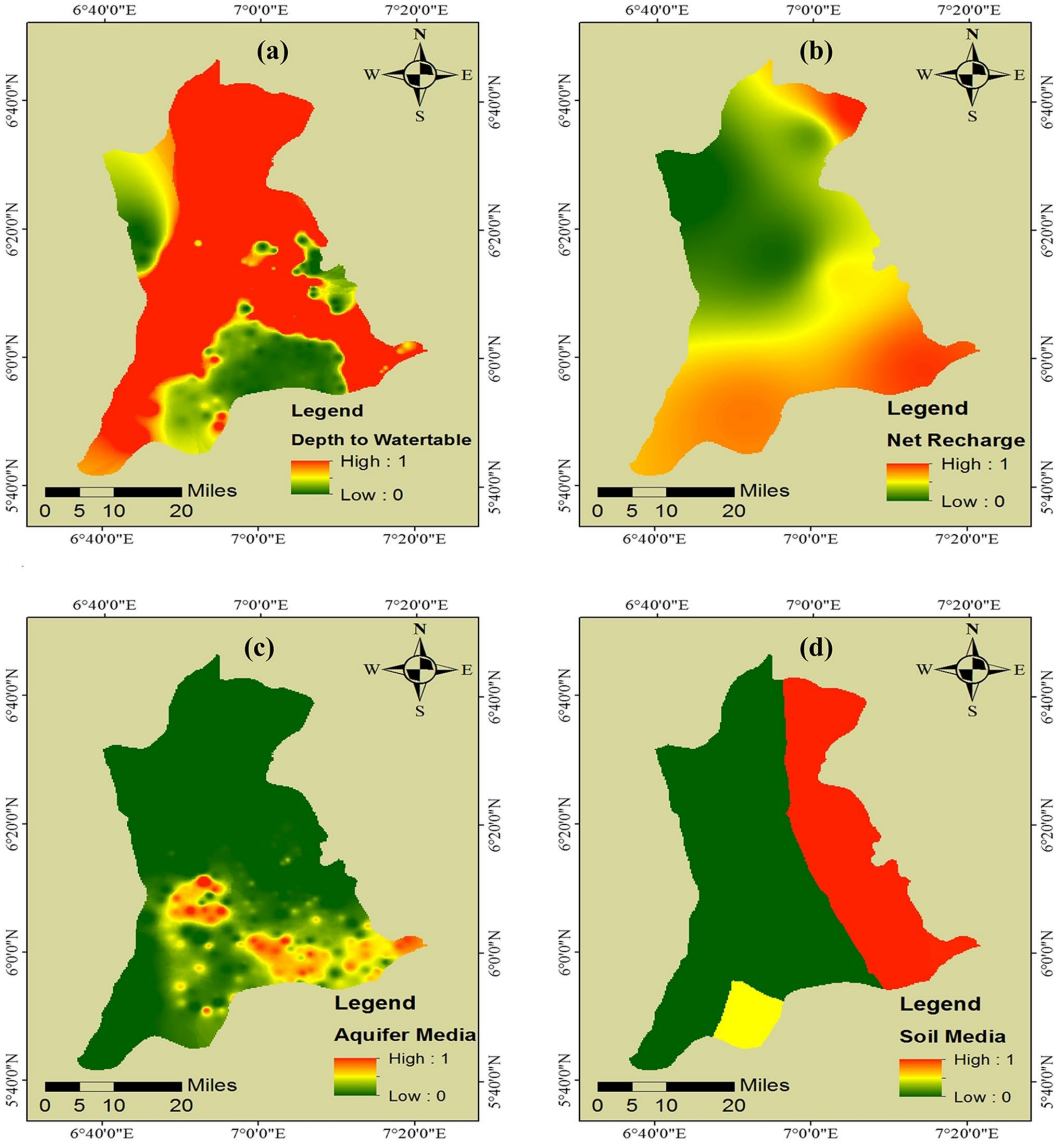

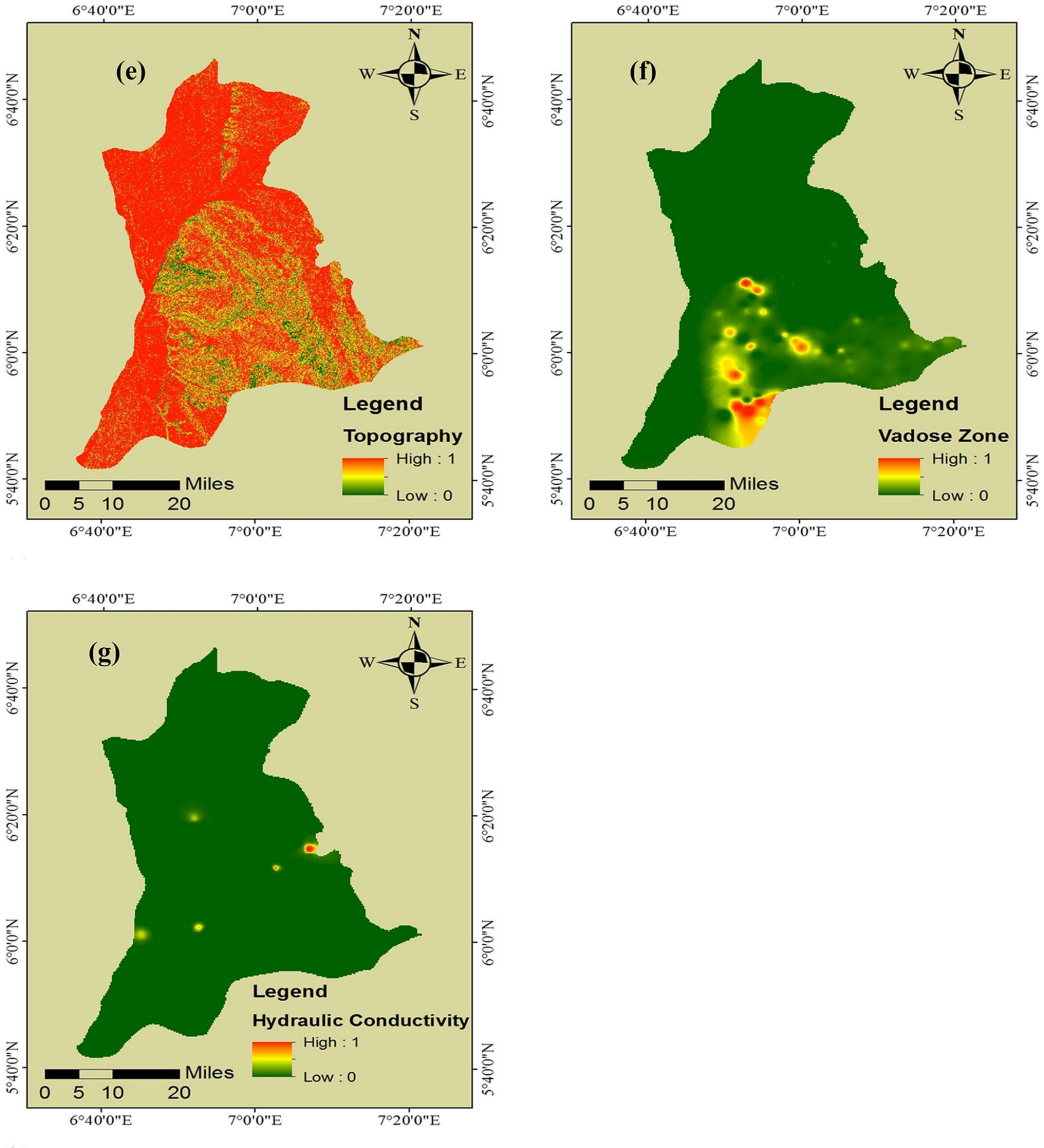
Delineated maps of hydrogeologic factors: (**a**) depth-to-water table; (**b**) net recharge; (**c**) aquifer media; (**d**) soil media; (**e**) topography; (**f**) vadose zone; and (**g**) hydraulic conductivity

The depth-to-water table captures the total distance covered by pollutants from the soil surface point of entry to the point of dissolution in the water table. As pollutants travel through soil media, attenuation significantly filters the pollutants through seepage (Bera et al. 2021). Hence, a greater depth-to-water table reduces groundwater pollution risks, as greater depth indicates higher pollutant travel time from the surface to the water table. The depth-to-water table in the study area ranged from 37.26 to 251.76 m, classified into 37.26–88.57 m, 88.58–110.44 m, 110.45–136.52 m, 136.53–172.69 m, and 172.70–251.76 m. Greater water depths were observed in Anambra West, Aguata, and Nnewi South LGAs, while lesser water depths were observed in Dunukofia, Oyi, and Idemili North and South LGAs. The fuzzy MS small membership function was used to standardize this factor as a lesser depth-to-water table poses more significant pollution risks. The result of the fuzzification is shown in Fig. 2a.

Net recharge, i.e., the total amount of water percolating soil media to recharge the water table (Moghaddam et al. 2018), is an essential factor in assessing groundwater vulnerability, as surface pollutants can easily dissolve and be transported in recharge water. In addition, more net recharge increases the propensity of pollutant dissolution, transport, and contamination of receiving water table (Lathamani et al. 2015; Bera et al. 2021). The net recharge obtained in the study area varied from 1 to 7 mm/year and was classified into 1–2.20 mm/year, 2.21–3.40 mm/year, 3.41–4.60 mm/year, 4.61–5.80 mm/year, and 5.81–7 mm/year. Based on this fact, the fuzzy large membership function was used to standardize this factor; see Fig. 2b.

An aquifer’s nature and flow rate are determined by its media, which also determines the route and path length of the pollutant (Bhuvaneswaran and Ganesh 2019). Furthermore, the aquifer media factor is crucial to salinity’s movement in groundwater as they provide interconnected pore spaces facilitating solute movement (Shakoor et al. 2020). Moreover, the pollution of an aquifer depends on the quantity and sorting of fine grains which make up the media. The finer the grains, the less the risk of the aquifer to pollution. The aquifer media in this study was modeled as the aquifer media resistivity values and ranged from 138.1 Ωm at a thickness of 42.13 m and a depth of 38.73 m to 8176.89 Ωm at a thickness of 24.86 m and a depth of 64.29 m. The resistivity values were further classified into five: 138.1–1209.94 Ωm, 1209.95–1966.53 Ωm, 1966.54–2817.70 Ωm, 2817.71–3700.39 Ωm, and 3700.4–8176.89 Ωm. The aquifer media factor was standardized using the fuzzy large membership function. Lower resistivity values were associated with more clayey and less permeable materials being restrictive to pollutant transport. The result of the fuzzification is shown in Fig. 2c.

The soil media (the topmost weathered layer above the vadose zone) significantly affects water table net recharge (Shah et al. 2021). It also impacts the pollutants’ mobilization and transport as a higher holding capacity of the soil media prolongs pollutants’ travel time (Hamza et al. 2014). There are five soil classes in the study area, dominated mainly by fluvisol, alluvial, and gleysol, with plinthosol and nitrosol occupying tiny parts of the study area. The soil classes were reclassified into numeric values ranging from 1–5, with 1 representing low risk and 5 representing high risk. Due to its high-water holding capacity and ability to delay pollutant migration, alluvial was assigned the numeric value of 1. In contrast, fluvisol was assigned the numeric value of 5 due to its weak topsoil formation. After reclassification, the soil media was standardized using the fuzzy large membership function, as shown in Fig. 2d.

In the DRASTIC model, the topography factor (which captures the impact of land surface slope) determines the propensity of pollutants to surface runoff or retention toward the water table (Tiwari et al. 2016). The slope of the study area ranged from 0° to 43.46°, with flat slopes dominating the northern part of the state. The slope was further classified into five: 0–1.36°, 1.37–3.58°, 3.59–6.48°, 6.49–11.25°, and 11.26–43.46°. The fuzzy MS small membership function was used to standardize this factor, as shown in Fig. 2e. This was conducted because flat slopes tend to retain water and its associated pollutants for more prolonged periods, whereas steep slopes encourage more runoff and less pollutant retention. Hence, there is a higher risk of pollutant infiltration and migration to the water table on flat slopes than on steep slopes.

The vadose zone (the unsaturated area between the ground surface and the water table) impacts the speed of pollutant migration into an aquifer (Saida et al. 2017). The type of material that constitutes the vadose zone influences groundwater pollution mechanisms, including biodegradation, mechanical filtration, sorption, volatilization, and dispersion (Bhuvaneswaran & Ganesh 2019). In addition, the vadose zone’s influence on groundwater’s vulnerability is a combination of topography and the aquifer media (Shakoor et al. 2020). In this study, the impact of the vadose zone factor was modeled as the resistivity values obtained from the vadose zone in the study area, ranging from 311.33 to 34,630.26 Ωm, which were further classified into 311.33–3675.93 Ωm, 3675.94–7309.70 Ωm, 7309.71–11,616.39 Ωm, 11,616.40–16,999.75 Ωm, and 16,999.76–34,630.26 Ωm. Previous studies considered various materials such as sand, gravel, sand-gravel, clay, and clay-sand (Ahada and Suthar 2018). Others excluded the impact of the vadose zone entirely due to the non-availability of data in the assessment of the zone (Adnan et al. 2018); since these materials have various resistivity values, the values of the resistivity of the zone were used in the classification of the zone (Bhatnagar et al. 2022). Higher resistivity values were observed in the southern part of the state, including Ihiala, Ekwusigo, Aguata, Idemili North, and South LGAs. The impact of the vadose zone factor was standardized using the fuzzy large membership function based on the same reason highlighted previously for the aquifer media factor. The result of the fuzzification is shown in Fig. 2f.

Hydraulic conductivity, an aquifer’s ability to transmit water, depends on a specific hydraulic gradient (Mondal et al. 2019; Vosoogh et al. 2017). This factor further indicates how fast pollutants travel, their residence time, and subsequent dilution ability (Yankey et al. 2021). Hydraulic conductivities in the study area ranged from 0.0124 to 0.7832 cm/s and were classified into 0.0124–0.1666 cm/s, 0.1667–0.3207 cm/s, 0.3208–0.4749 cm/s, 0.4750–0.6291 cm/s, and 0.6292–0.7832 cm/s. Most parts of Anambra state had low hydraulic conductivity, but high hydraulic conductivity was also observed in some parts of Awka North and South LGAs. The hydraulic conductivity factor was standardized using the fuzzy large membership function, and the result is shown in Fig. 2g. The choice of fuzzification was based on the correlation of higher conductivities with higher pollution risk (Arya et al. 2020).

### Determination of the relationship between hydrogeologic factors

A hybrid MCDM model of IRN, DEMATEL, and ANP methods was used to evaluate the interrelationship between the hydrogeologic factors of groundwater pollution and subsequently determine their individual weights. Through the expert survey, a pairwise comparison matrix was acquired and processed using the IRN method to eliminate uncertainty and vagueness in the experts’ decisions. The matrix obtained contained a pair of real numbers, and these values were transformed to interval rough numbers using Eqs. (2) to (12). Equations (2) and (12) were further employed to aggregate the decision of the experts and obtain our IRN decision matrix. The interval rough numbers contained in the IRN decision matrix were then transformed to crisp numbers using Eqs. (2) and (12) to aid data interpretation. This transformation gives us our initial decision matrix $D$, as shown in Table 2.

**Table 2:** Initial decision matrix

j	D	R	A	S	T	I	C
i							
D	0.00	4.00	3.00	3.00	2.52	2.92	2.52
R	4.32	0.00	3.54	3.00	3.50	3.78	3.54
A	3.00	3.50	0.00	3.56	1.84	2.83	4.56
S	3.00	3.00	3.56	0.00	3.00	3.36	4.23
T	2.80	3.50	2.00	3.00	0.00	1.80	3.00
I	2.92	3.78	2.83	3.36	1.80	0.00	3.20
C	2.52	3.54	4.56	4.23	3.00	3.36	0.00

The elements of the matrix $D$ were normalized using Eqs. (19) to (21), thereby obtaining the matrix $\overline{\overline{Z}}$. The total relationship matrix $T,$ which illustrates the interrelationships between the hydrogeologic factors, was derived from the matrix $\overline{\overline{Z}}$ using Eqs. (22) and (23). The resultant matrix $T$ is shown in Table 3.

**Table 3:** Total relationship matrix

j	D	R	A	S	T	I	C
i							
D	0.9686	1.2396	1.1409	1.1585	0.9391	1.0714	1.1853
R	1.2963	1.2663	1.3300	1.3337	1.1123	1.2595	1.4005
A	1.1605	1.3033	1.1012	1.2595	0.9780	1.1418	1.3392
S	1.1874	1.3177	1.2687	1.1478	1.0420	1.1852	1.3582
T	0.9946	1.1238	1.0139	1.0650	0.7615	0.9445	1.1033
I	1.0897	1.2353	1.1408	1.1759	0.9161	0.9574	1.2134
C	1.2228	1.3923	1.3564	1.3661	1.0855	1.2350	1.2539

**Fig. 3:**
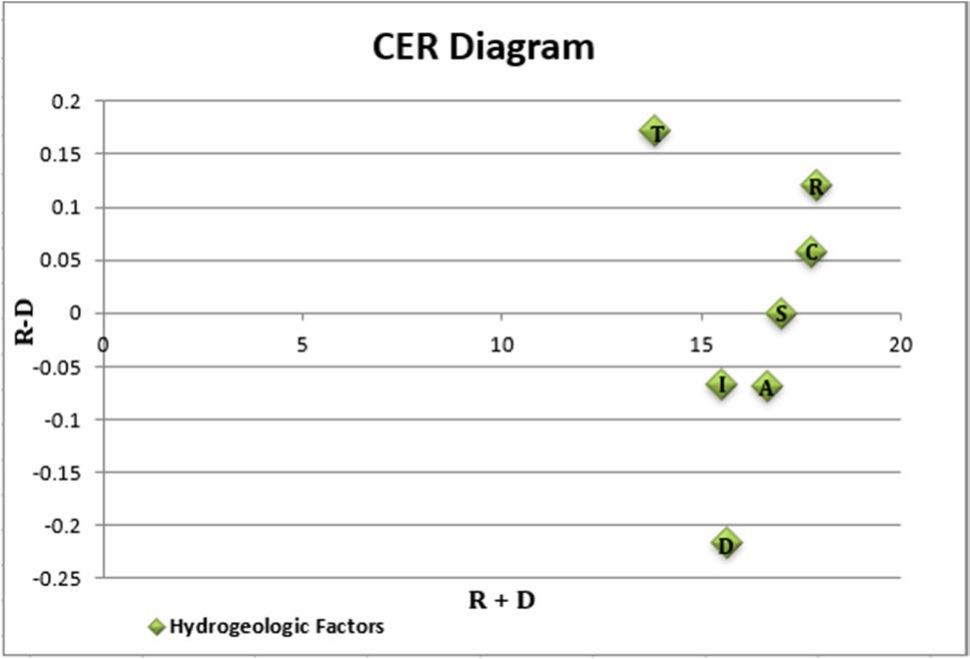
Cause-and-effect relationship (CER) diagram of hydrogeologic factors

Each row of the matrix $T$ was summed up as indicated by Eq. (24) to obtain our $R$ factor, and each column was also summed up as indicated by Eq. (25) to obtain the $S$ factor. Determining $R$ and $S$ factors is necessary to examine each hydrogeologic factor’s significance and its effect on groundwater vulnerability. The values of $R$ and $S$ describe each hydrogeologic factor’s indirect and direct effects from and transferred to other hydrogeologic factors. The result for $R$, $S$, $R+S$, and $R-S$ factors are shown in Table 4. The $R+S$ and $R-S$ factors were then used to construct the CER diagram and visualize the complicated interrelationship between the hydrogeologic factors, where the *x*-axis contains values of the $R+S$ factor and the *y*-axis contains values of the $R-S$ factor (see Fig. 3).

**Table 4:** Values of importance and prominence

	$R$	$S$	$R+S$	$R-S$
D	7.7034	7.9199	15.6233	− 0.2165
R	8.9986	8.8783	17.8769	0.1203
A	8.2835	8.3519	16.6354	− 0.0684
S	8.5070	8.5065	17.0135	0.0005
T	7.0066	6.8345	13.8411	0.1721
I	7.7286	7.7948	15.5234	− 0.0662
C	8.9120	8.8538	17.7658	0.0582

The interpretation of the result obtained for the $R-D$ factor shows that net recharge, soil media, topography, and hydraulic conductivity are influential hydrogeologic factors and thus categorized as “causes.” The result also indicates that depth-to-water table, aquifer media, and impact of the vadose zone factors are influenced by other factors and thus categorized as “effects.” Furthermore, the topography factor was given the highest priority with the highest positive value of 0.1721, and the depth-to-water table factor was given the lowest priority with the highest negative value of − 0.2165. The priority given to topography can be attributed to its role in the penetration of water recharge and subsequent migration of associated pollutants. By influencing the penetration of water recharge, topography affects the amount of water passing through the water table to the aquifer media.

### Determination of individual weights of hydrogeologic factors

The individual weights of hydrogeologic factors were determined using the ANP method, and the total relationship matrix was employed as input data. Expert opinion was relied upon to arrive at an optimum α threshold value of 1.08. This was used to filter out minor influences from the matrix $T$ to obtain an unweighted supermatrix, i.e., the elements of the matrix $T$ with values less than the α value of 1.08 were equated to 0 to obtain the unweighted supermatrix ${T}^{\propto }$ as illustrated by Eq. (26). The matrix ${T}^{\propto }$ was then normalized using Eq. (27), and the resultant matrix, which is our weighted supermatrix $\widetilde{W}$ is shown in Table 5. The final step in this phase involved the determination of the weight of the hydrogeologic factors from the weighted supermatrix $\widetilde{W}$. To achieve this, the matrix $\widetilde{W}$ was limited by raising it to the power of 13. This produced convergent values representing the individual weights of the hydrogeologic factors; the result is shown in Table 6. The resultant individual weights of the hydrogeologic factors, as determined by the ANP method, indicate that the net recharge factor has the highest weight of 0.1986, followed closely by the hydraulic conductivity factor with a weight of 0.1969. In descending order, the soil media, aquifer media, impact of the vadose zone, depth-to-water table, and topography factors weighted 0.1644, 0.1609, 0.1248, 0.1047, and 0.0497, respectively. This result shows that hydrogeologic factors, net recharge, and hydraulic conductivity constitute the two most significant factors to consider when evaluating groundwater vulnerability in the study area.

**Table 5:** Weighted supermatrix

	D	R	A	S	T	I	C
D	0.0000	0.1396	0.1555	0.1557	0.0000	0.0000	0.1339
R	0.2176	0.1426	0.1812	0.1792	0.5061	0.2612	0.1582
A	0.1948	0.1468	0.1501	0.1693	0.0000	0.2368	0.1513
S	0.1993	0.1484	0.1729	0.1542	0.0000	0.2458	0.1534
T	0.0000	0.1266	0.0000	0.0000	0.0000	0.0000	0.1246
I	0.1829	0.1391	0.1555	0.1580	0.0000	0.0000	0.1370
C	0.2053	0.1568	0.1848	0.1836	0.4939	0.2561	0.1416

**Table 6:** Individual weights of hydrogeologic factors

Hydrogeologic factor	Final weight
Depth-to-water table (*D*)	0.1047
Net recharge (*R*)	0.1986
Aquifer media (*A*)	0.1609
Soil media (*S*)	0.1644
Topography (*T*)	0.0497
Impact of vadose zone (*I*)	0.1248
Hydraulic conductivity (*C*)	0.1969

### Production of groundwater vulnerability map

**Fig. 4:**
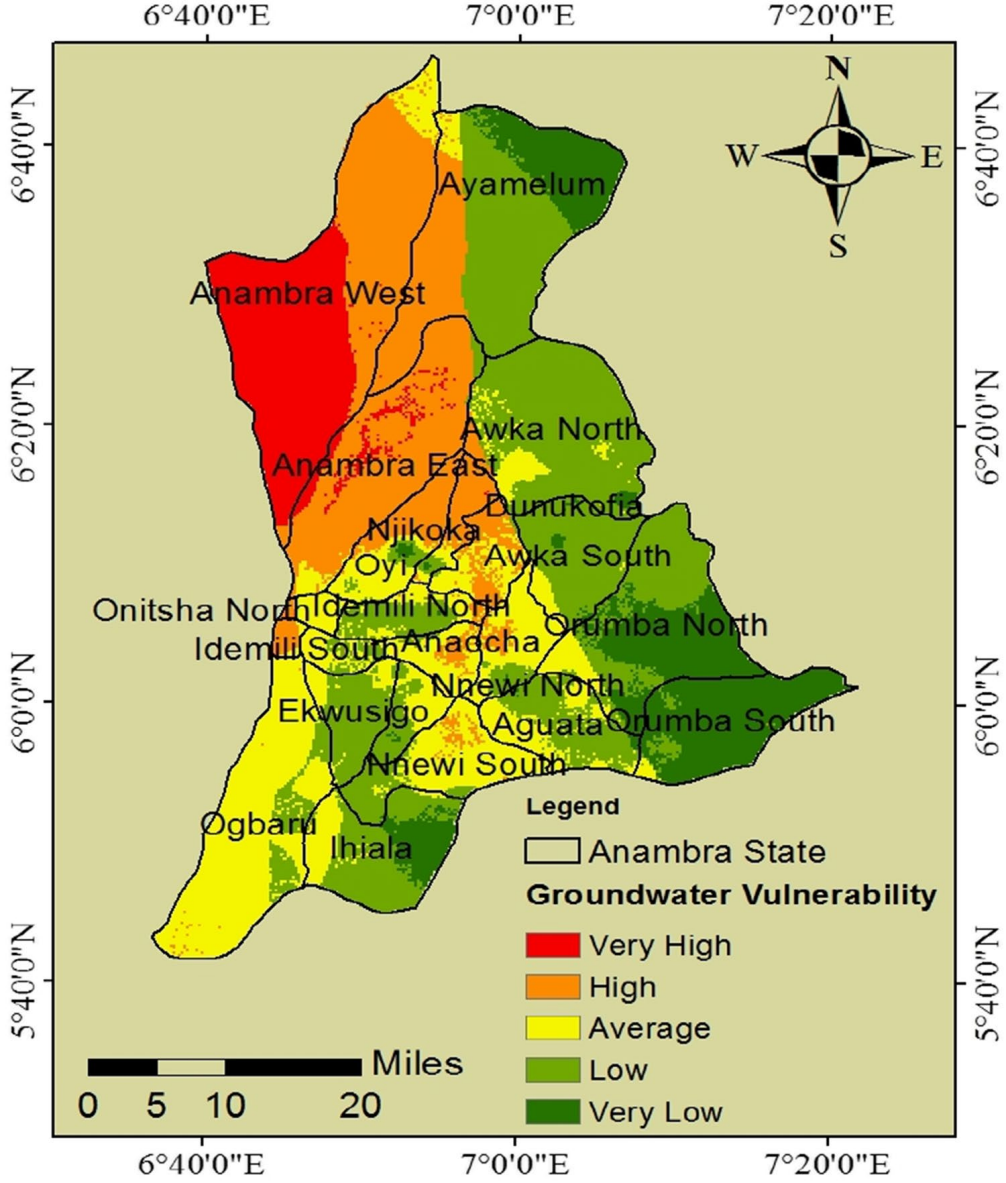
Groundwater vulnerability distribution of the study area

The DRASTIC method generated a spatial distribution of groundwater vulnerability in the study area. The fuzzified map layers of the hydrogeologic factors represent the model’s rating factor, and the IRN-DEMATEL-ANP weight of the hydrogeologic factors represents the weighting factor of the model. The raster calculator function was used to run the DRASTIC model, and the obtained result is shown in Fig. 4. The resultant groundwater vulnerability map was classified into five distinct categories: “very high,” “high,” “average,” “low,” and “very low.” The resultant groundwater vulnerability maps are presented at the local government areas (LGA) levels of the state to elucidate variations in geographical locations. The study area has an estimated land mass of about 4563 km^2^, with the resultant groundwater vulnerability map showing that 12.98% (592.21 km^2^) of that land mass falls into the very low vulnerability class, 31.90% (1455.78 km^2^) falls into low vulnerability class, 23.52% (1073.20 km^2^) falls into average vulnerability class, 21.75% (992.42 km^2^) falls into high vulnerability class, and 9.85% (449.40 km^2^) falls into very high vulnerability class. Anambra West and East LGA were classified as highly vulnerable areas; this agrees with previous studies on flooding, indicating that the study area’s western region is prone to flooding (Chukwuma et al. 2021). The critical vulnerability observed in some parts of the study area can be attributed to heavy rainfall recorded in those areas and the associated large volumes of net recharge, which tends to carry pollutants and leach them into groundwater sources; this is usually linked to flooding. Another reason for the critical vulnerability is high hydraulic conductivity which leads to an intensified rate of groundwater movement and associated pollutants to the aquifer.

This study is critical for groundwater resource sustainability; several researchers with various reports have strongly recommended integrating a hybrid decision model with a geospatial-based tool. A recent study integrated hydrogeological, geospatial, and multi-criteria decision tools in groundwater management; the study attempted to classify groundwater recharge potential zones in northern Maharashtra, India (Sahu et al. 2022). The study applied an analytical hierarchy process model and successfully categorized the groundwater recharge zones. The study classified the zones into high, moderate, and low recharge zones. In contrast, this study classified the area into five zones, similar to previous studies that also applied a modified DRASTIC model. Several studies have emphasized on spatial variability of groundwater to pollution using the DRASTIC model; a study on groundwater vulnerability in the Pesshar District of Pakistan reported that the southern and western parts of the study areas indicated low vulnerability, whereas the south-eastern and north-eastern parts indicate moderate vulnerability (Adnan et al. 2018). For this study, very high, high, and average vulnerability classes dominated LGAs such as Orumba North, Orumba South, Ihiala, Ayamelum, and Awka South and Awka North. In Orumba North and South LGAs, the total area was classified as less susceptible (very low, low, and average) to pollution. A similar result was observed in Awka North and South, where the state’s capital is located, with 96.7% and 100% of the respective total areas of Awka North and Awka South classified under low vulnerability.

Studies on the integration of decision models with the geospatial application have several advantages owing to the unique capacity of GIS applications in managing geospatial data and the ability of the MCDM model to handle complex decision-making problems (Haroon and Muhammad 2022). This study is valuable for groundwater resource management; areas of higher vulnerability from this study should receive greater interventions, resource allocation, and risk management to prevent groundwater pollution. Generally, higher resource allocation and priority to risk should be given to higher-risk zones such as Anambra West and East LGA, considering the region’s high vulnerability to groundwater pollution.

Identification of factors and ranking of these factors is critical in the management of groundwater quality. This study further suggests that net recharge is the major hydrological factor affecting groundwater vulnerability determination. Owing to the factor’s significance, special attention should be given to regions that experience high surface pollution due to agricultural and industrial activities in the study area. This is important, as such activities would increase surface pollution and net recharge, which may increase the chances of these pollutants reaching the water table (Lathamani et al. 2015). Therefore, factor rating is a critical evaluation strategy in groundwater quality assessment. The fuzzified map layers of the hydrogeologic factors represent the rating factor for this study; this is similar to several research works that successfully attempted to rate factors that contribute to groundwater quality. For example, Bouselsal and Saibi (2022) assessed geochemical characteristics and groundwater quality using standardized factor scores to represent the impact of processes on water quality. Similarly, a study by Balaji et al. (2021) improved both the rates and weights in the original DRASTIC model by applying a modified DRASTIC model using a metaheuristic algorithm approach. However, in this study, the IRN-DEMATEL-ANP weight of the hydrogeologic factors, representing the weighting factor of the model, was optimized, resulting in a significant improvement.

### Validation of the groundwater vulnerability assessment model

**Fig. 5:**
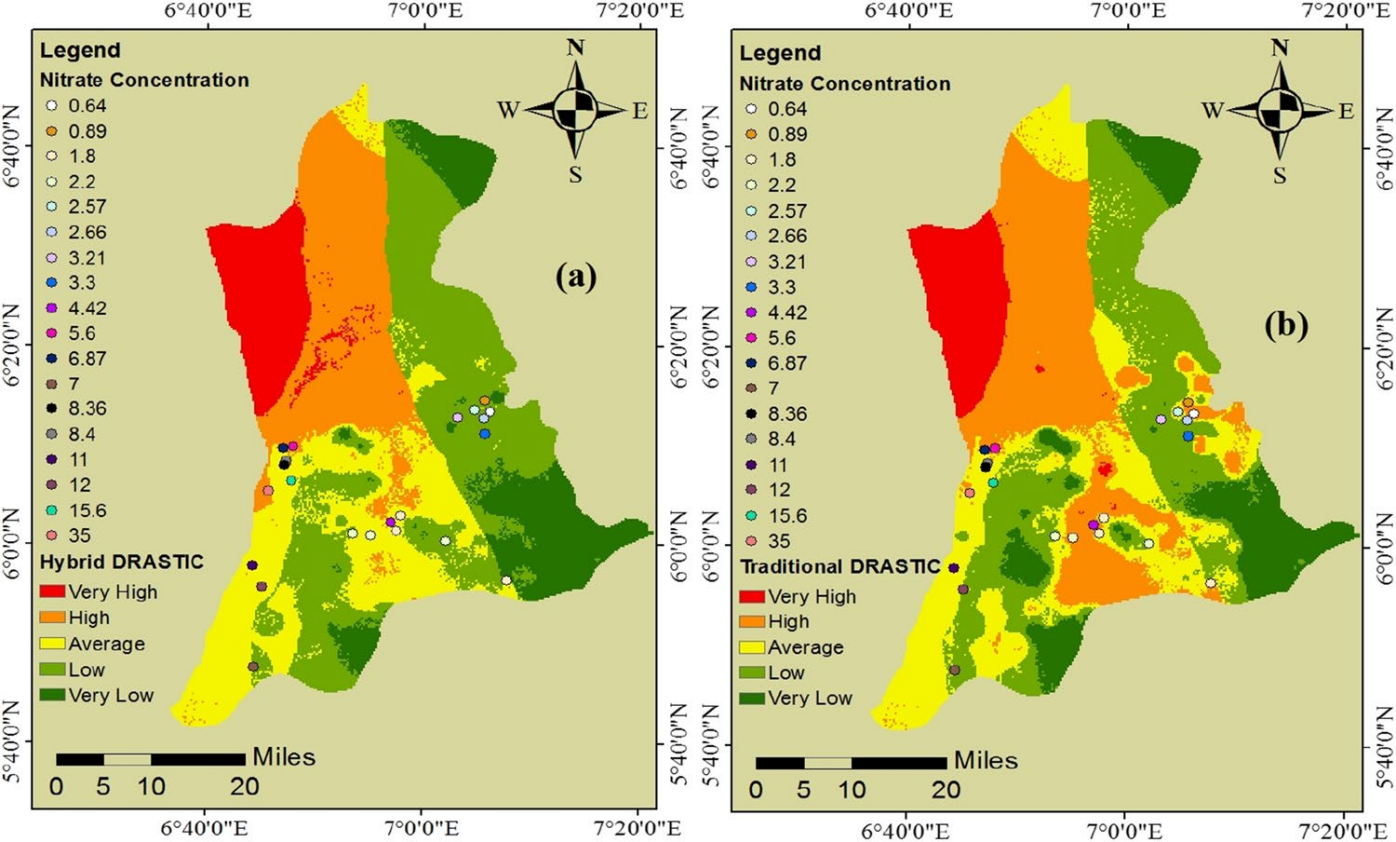
(**a**) Validation map of the hybrid DRASTIC model; (**b**) validation map of the traditional DRASTIC model

In this study, nitrate concentration was considered a significant source of groundwater pollution and used to validate our assessment model compared to the traditional DRASTIC model. This is per previous research work (Moghaddam et al. 2022; Bouselsal and Saibi 2022; Haroon and Muhammad 2022). The decision to use nitrate concentration was based on data availability and the high rate of agricultural and industrial activities in the study area. As shown in Fig. 5a, the obtained nitrate concentrations from 22 boreholes in the study area were overlaid with our resultant vulnerability map to determine the accuracy of the map. Most of the nitrate concentrations obtained agreed with the different zones of vulnerability classified using our hybrid MCDM assessment model.

Out of 10 boreholes with a higher concentration of nitrate (> 4 mg. L^−1^), the vulnerability map based on our hybrid MCDM model classified six under the high vulnerability class and four under the average vulnerability class. Out of 12 boreholes with a lower concentration of nitrate (> 4 mg. L^−1^), ten were classified under low vulnerability, and the remaining two were under average vulnerability class. In comparison, the traditional DRASTIC model ranked 4 out of 10 higher concentrations under high vulnerability, four under average vulnerability and two under low vulnerability classification. For the lower concentrations, the traditional DRASTIC model classified 6 out of 12 under low vulnerability, two under average vulnerability and four under high vulnerability. See Supplementary information 1 for the data for the nitrate concentrations and the geographical coordinate. For instance, the borehole located at 6.106379 and 6.797428 latitude and longitude, respectively, with a nitrate concentration of 15.6 mg. L^−1^ was under the average classification of the hybrid model. At the same time, it was classified in the traditional model within the low vulnerability zone. Generally, the hybrid model shows better agreement with the nitrate concentration, an indication of improvement in vulnerability assessment, which is critical in groundwater resource management.

Based on these results, the hybrid MCDM model performed better than the traditional MCDM model. Though not a robust method of validating groundwater vulnerability results, nitrate concentrations help define sources of surface pollution and the manner of the permeable media’s response to the contamination process (Aydi 2018). A recent study asserted that the original DRASTIC model weights and rates indicate a poor correlation between groundwater vulnerability index and nitrate concentration. This can be attributed to the tendency to consider each factor’s relative significance and subsequent weight, as also established by several researchers (Saida et al. 2017; Balaji et al. 2021). However, this study’s improved DRASTIC model performance due to a novel combination of hybrid models indicates that the approach used here is veritable in groundwater pollution assessment.

## Conclusion

The vulnerability of groundwater resources to pollution has been assessed in this study. A modified DRASTIC method was employed for the assessment. To modify and improve the DRASTIC method, this study employed hybrid MCDM models of IRN, DEMATEL, and ANP methods to determine the weights and ratings of the hydrogeologic factors of the DRASTIC method. The application of GIS was utilized to delineate the thematic maps of the hydrogeologic factors and run the model. The hybrid MCDM model proved effective and efficient in evaluating the factors’ interrelationships and assigning relative weights based on expert surveys. The DEMATEL method found the topography to be the most influential. The ANP method found the net recharge factor to be the most significant factor in the study area and the topography factor to be the least significant. The obtained vulnerability map indicates that the study area is quite vulnerable to groundwater pollution, with about 68% of the total study area falling between very high and average vulnerability classes. Based on obtained nitrate concentrations, the assessment model adopted in this study was validated compared to the traditional DRASTIC method, and the hybrid model showed better predictive performance than the traditional DRASTIC method. However, it is recommended that the adopted method in this study should be investigated further by considering anthropogenic factors in addition to the hydrogeologic factors of the DRASTIC method. The role of the hybrid integration of MCDM models of IVFRN, DEMATEL, and ANP methods in modifying the DRASTIC model for improved assessment of groundwater pollution is demonstrated in this study. While the obtained nitrate concentrations in the study area are well within the permissible limits of the WHO, there is still a need to put appropriate groundwater resource management practices in place, especially in those areas with high vulnerability. Most of these highly vulnerable areas are predominantly agricultural areas, and agricultural activities are a major source of surface pollution and the subsequent migration of pollutants to groundwater. Agricultural activities such as fertilizer application in these areas with high vulnerability should be properly monitored and regulated.

## Supplementary Information

Below is the link to the electronic supplementary material.

Supplementary file1 (DOCX 28 KB)

## Data Availability

In addition to the supplementary material, all other data and materials are available upon request.
