# Supplementary material for: Groundwater vulnerability to pollution assessment: an application of geospatial techniques and integrated IRN-DEMATEL-ANP decision model
Source: Environ Sci Pollut Res Int. 2023 Feb 14;30(17):49856–74. doi: 10.1007/s11356-023-25447-1 (PMC10104943; doi:10.1007/s11356-023-25447-1)
Supplement: Supplementary file 1 — Supplementary file1 (DOCX 28 KB) [file 11356_2023_25447_MOESM1_ESM.docx]

**Groundwater vulnerability to pollution assessment: An application of geospatial techniques and integrated IRN-DEMATEL-ANP decision model**

**AUTHORS**

**^1, 4^**Emmanuel Chibundo Chukwuma, **^1^**Chris Chukwuma Okonkwo, **^2*^**Oluwasola Olakunle Daniel Afolabi, **^3^**Quoc Bao Pham, **^1^**Daniel Chinazom Anizoba, **^1^**Chikwunonso Divine Okpala.

**AFFILIATIONS**

**^1^** Department of Agricultural and Bioresources Engineering, Faculty of Engineering, Nnamdi Azikiwe University, Awka, Nigeria

**^2^** School of Architecture, Building and Civil Engineering, Loughborough University, Loughborough, LE11 3TU, UK.

**^3^** Faculty of Natural Sciences, Institute of Earth Sciences, University of Silesia, Katowice, Będzińska street 60, 41-200, Sosnowiec, Poland

^4^ Research Fellow, Future Africa, University of Pretoria, South Africa

**CORRESPONDING AUTHOR’S EMAIL:** [o.o.d.afolabi2@lboro.ac.uk](mailto:o.o.d.afolabi2@lboro.ac.uk)

**ACKNOWLEDGEMENT**

This research did not receive any specific grant from funding agencies in the public, commercial, or not-for-profit sectors. The views expressed in this paper are those of the authors.

**Supplementary Information 1: Nitrate Concentration at various locations in the study area**

| **Boreholes** | **Latitude** | **Longitude** | **Nitrate Conc. (mg. L^-1^)** |
| --- | --- | --- | --- |
| 1 | 6.036698 | 6.950176 | 4.42 |
| 2 | 6.014717 | 6.919309 | 2.2 |
| 3 | 6.017523 | 6.892651 | 2.2 |
| 4 | 6.005363 | 7.035294 | 2.2 |
| 5 | 5.939888 | 7.129766 | 1.8 |
| 6 | 6.047922 | 6.966077 | 1.8 |
| 7 | 6.023135 | 6.95953 | 1.8 |
| 8 | 5.964675 | 6.73738 | 11 |
| 9 | 5.928663 | 6.752346 | 12 |
| 10 | 5.793503 | 6.739251 | 7 |
| 11 | 6.089499 | 6.760764 | 35 |
| 12 | 6.2125 | 7.054002 | 3.21 |
| 13 | 6.210629 | 7.094222 | 2.66 |
| 14 | 6.185374 | 7.095158 | 3.3 |
| 15 | 6.163861 | 6.799582 | 5.6 |
| 16 | 6.140009 | 6.788825 | 8.4 |
| 17 | 6.132345 | 6.785995 | 8.36 |
| 18 | 6.16067 | 6.7843 | 6.87 |
| 19 | 6.106379 | 6.797428 | 15.6 |
| 20 | 6.241029 | 7.09469 | 0.89 |
| 21 | 6.22466 | 7.079257 | 2.57 |
| 22 | 6.222789 | 7.103576 | 0.64 |
